# Rapid Epidemiological Assessment of Onchocerciasis in a Tropical Semi-Urban Community, Enugu State, Nigeria

**Published:** 2013

**Authors:** JE Eyo, GC Onyishi, CU Ugokwe

**Affiliations:** Department of Zoology and Environmental Biology, University of Nigeria, Nsukka, Enugu State, Nigeria

**Keywords:** Onchocerciasis, River blindness, *Onchocerca volvulus*, Prevalence, Nigeria

## Abstract

**Background:**

This study was carried out in Opi-Agu a tropical semi-urban autonomous community comprising of three villages in Enugu State, Nigeria, between the months of April and June 2010. It was designed to determine the prevalence of *Onchocerca volvulus* infection and assess the perception of the disease among the inhabitants of this community.

**Methods:**

A total number of 305 individuals comprising of 148 males and 157 females were examined for various manifestations of onchocerciasis symptoms using rapid epidemiological assessment (REA) method.

**Results:**

Out of this number, 119 (39.02%) individuals were infected. Prevalence of infection among age groups and villages varied. Age group 41 yr and above had the highest (31.00%) prevalence, while among the villages, Ogbozalla village ranked higher (45.71%) than the other villages. Overall the prevalence of infection among the sexes revealed that males were more infected (43.24%) than the females (35.03%). Lichenified onchodermatitis (LOD) was the most prevalent (35.29%) onchocerciasis symptom among others identified in the area, while leopard skin (LS) had the lowest (20.17%) occurrence and blindness (0.00%) which is the most devastating effect of *O. volvulus* infection was not observed. Questionnaire responses from 410 individuals revealed that 34.8% respondent from Idi village and 28.1% from Ibeku village believed that *O. volvulus* infection occurs through poor personal hygiene. Bite of blackfly ranked least (10.6%) among the respondent's knowledge of the causes of onchocerciasis in Opi-Agu community.

**Conclusion:**

Opi-Agu community members had poor knowledge of onchocerciasis, the vector and of its etiologic organism. There is need for integration of community health education with mass chemotherapy.

## Introduction

Onchocerciasis, commonly called river blindness is a disease that results from infection with the filarial nematode known as *Onchocerca volvulus*. The parasite is transmitted to humans who are the only known vertebrate host through the bite of a female blackfly of the genus *Simulium*, and in Nigeria it is transmitted by *S. damnosum* complex (1, 2). *O. volvulus* infection is presently endemic in Africa, Latin America and Yemen where as at 1997 estimates of 123 million people were at risk of infection while about 17 to 18 million people are already infected (3). It was also estimated that approximately 95% of all infected people live in Africa particularly in the sub–Saharan region (4).

Onchocerciasis is seen as debilitating disease of great magnitude causing more blindness than any other disease. In most endemic areas, more than one third of the adult population is blind. An estimate of 600,000 people worldwide are blind due to the disease and about 1.5 million people are severely impaired visually (5).

There is a wide spectrum of onchocercal clinical manifestations which include severe itching, alteration of skin pigmentation with areas of hyper and hypopigmentation such as leopard skin, lizard skin (6), onchodermatitis, palpable noodles, hanging groin, lymphadenopathy, scrotal involvement such as hydrocele and genital elephantiasis (7). Onchocerciasis constitutes a major public health problem and has hindered economic growth in endemic areas. The threat of the disease had led to the abandonment of more than 250,000 sq km of useful land in West Africa (8). The disease also reduces self esteem, concentration and marriage prospects for sufferers. It causes decreased productivity and impoverishes the infected individuals. Many research works on *O. volvulus* infection have been carried out in different parts of Nigeria and in Enugu State which include the works of Edungbola (9), Gemade and Utsalo (10), Edungbola *et al*. (11), Ubachukwu and Anya (12, 13) and a host of others but non had been documented on Opi-Agu, Nsukka, Enugu State, Nigeria, which has an environment that is conducive for the disease to thrive.

This study aimed at: (i) assessing the prevalence of *O. volvulus* infection in Opi-Agu community, (ii) determining the various manifestations of onchocercal skin diseases (OSD) and palpable nodules and (iii) assessing the perception of this disease by community members.

## Materials and Methods

**Fig. 1:**
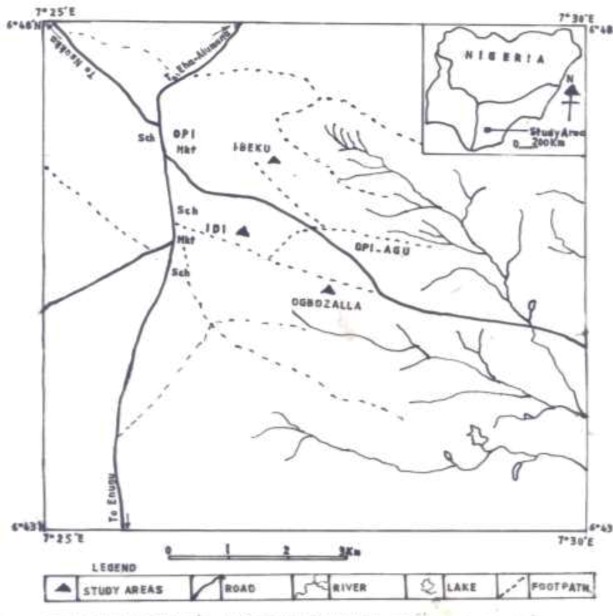
Map of Opi-Agu autonomous community showing the study area. Source: Federal Surveys of Nigeria (14), Nsukka sheet 287, northeast and southeast

Opi-Agu is a tropical semi-urban autonomous community comprising of three large villages located within latitude 7° 25’ to 7° 30’ E and longitude 6° 43’ to 6° 48’ N (Fig. 1) (14). The three villages comprises of about fifty three hamlets. It is intersected by numerous green hills and valleys. The vegetation falls within the rainforest-savanna mosaic and the climate is tropical. The people are predominantly farmers and cultivate crops like yam, cocoyam, maize, legumes and vegetables. The community is also endowed with streams and rivers which serve for domestic, recreational and agricultural purposes.

**Fig. 2:**
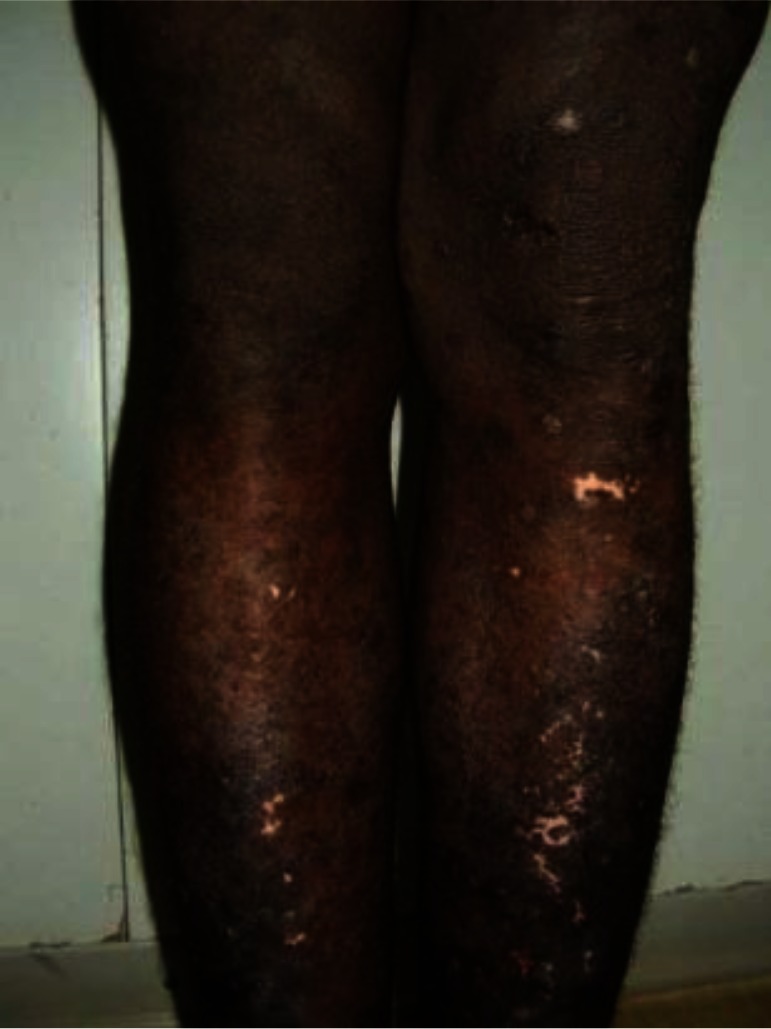
Lichenified onchodermatitis in a young male. Source: Bari and Rahman (19).

**Fig. 3:**
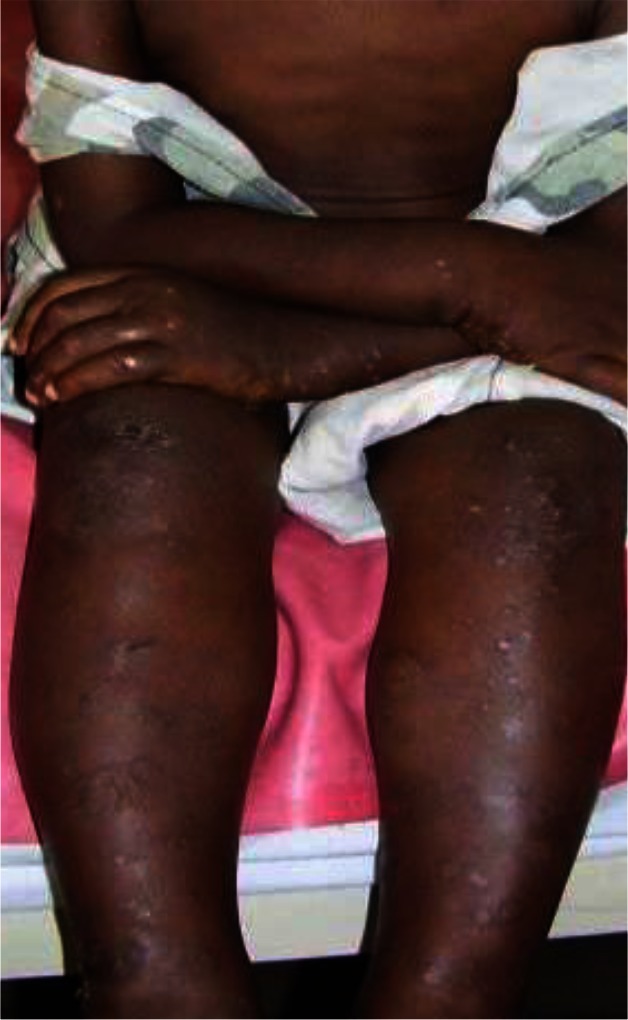
Maculo-papular rashes (MPR) in a young boy. Source: Bari and Rahman (19).

**Fig. 4:**
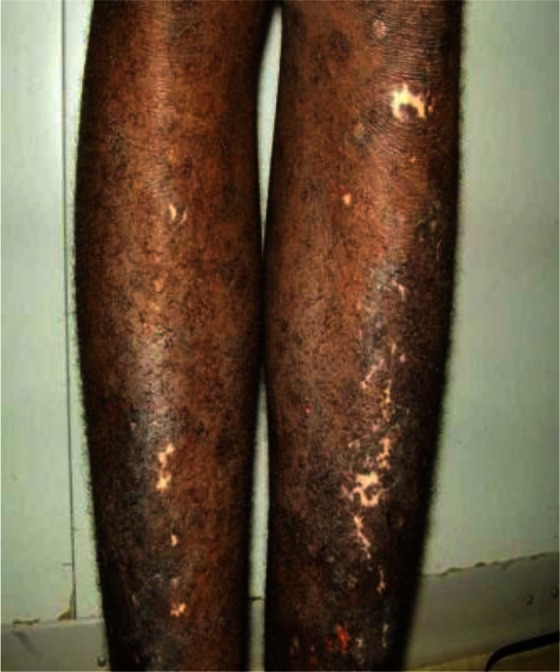
Leopard skin in a young male. Source: Bari and Rahman (19).

**Fig. 5:**
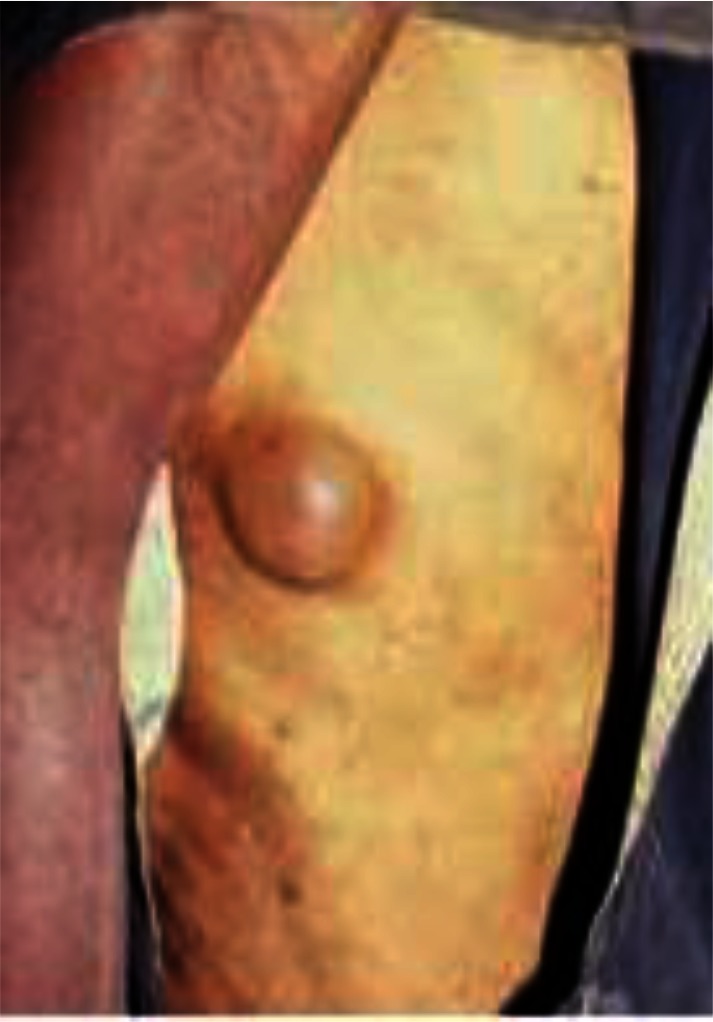
Palpable nodules in a young male. Source: Amazigo and Leak (18).

**Sampling:** Random sampling using lottery method was employed for the selection of 305 subjects (Idi village 93, Ogbozala village 105 and Ibeku village 107) used for the study (15) between the months of April and June 2010. Prior to the study, ethical clearance was obtained from Enugu State Ministry of Health and permission to carry out the research was obtained from the community leader and village heads. The people were also enlightened on the need for the study. Clinical examination of the subjects was carried out using rapid epidemiological assessment (REA) method (15) using the services of five nurses from the community health centers. The disease visible symptoms such as onchocercal blindness (OB), lichenified onchodermatitis (LOD) (Fig. 2), maculopapular rashes (MPR) (Fig. 3), leopard skin (LS) (Fig. 4) and palpable nodules (Fig. 5) were searched for. Questionnaires were also administered to assess the peoples’ perception of the disease.

Data generated were subjected to simple percentages to determine the prevalence of infection among the villages, age groups, sexes and to assess the perception of the disease among the community members. The distribution of onchocercal skin diseases (OSD) among the various age groups were also assessed using percentages.

## Results

A total of 305 individuals comprising of 148 males and 157 females were examined for onchocerciasis symptoms. One hundred and nineteen (39.02%) persons were infected. Among the villages studied, Idi village had the lowest prevalence (26.88%) while Ogbozalla village and Ibeku village had (45.71%) and (42.99%) prevalences, respectively (Table 1). A progressive increase in prevalence was observed among the age groups examined.

**Table 1:** Community and age prevalence of onchocerciasis in Opi-Agu a tropical semi-urban community in Enugu State, Nigeria

Community	Number Examined	Number Infected	Prevalence Percentage	Age groups (yr)				

				9 – 16	17 – 24	25 – 32	33 – 40	41 & above
**Idi Village**	93	25	26.88	3(12.00)	4(16.00)	8(32.00)	7(28.00)	3(12.00)
**Ogbozala Village**	105	48	45.71	4(8.33)	9(18.75)	12(25.00)	10(20.83)	13(27.08)
**Ibeku Village**	107	46	42.99	3(6.52)	5(10.87)	7(15.23)	10(21.74)	21(45.65)
**Total**	305	119	39.02	10(8.40)	18(15.13)	27(22.69)	27(22.69)	37(31.09)

The highest (31.09%) prevalence was observed within age group 41 and above while the least (8.40%) prevalence occurred within age group 9 – 16 years. Age group 25 – 32 years and 33 – 40 years had a prevalence of (22.69%), respectively (Table 1). Overall Prevalence of onchocerciasis symptoms among the sexes showed that the males were more infected than the females. Out of the 148 males examined, 64(43.24%) were positive for *O. volvulus* infection while the females had 55(35.03%) prevalence (Table 2). The most prevalent onchocercal sign in Opi-Agu community was lichenified onchodermatitis (LOD) which was highest (48.65%) among age groups 41 and above (Table 3).

**Table 2:** Gender and age prevalence of onchocerciasis in Opi-Agu a tropical semi-urban community in Enugu State, Nigeria

Age Group (yr)	Male			Female		

	Number examined	Number infected	Prevalence percentage	Number examined	Number infected	Prevalence percentage
**9 – 16**	21	8	38.09	18	2	11.11
**17 – 24**	28	11	39.85	29	7	24.14
**25 – 32**	34	15	44.12	37	12	32.43
**33 – 40**	31	14	45.16	34	13	38.24
**41 & above**	34	16	47.06	39	21	53.85
**Total**	148	64	43.24	157	55	35.03

**Table 3:** Symptomatic and age prevalence of onchocerciasis in Opi-Agu a tropical semi-urban community in Enugu State, Nigeria

Age Group (yr)	Number examined	Number positive	Prevalence (%) of onchocercal symptoms				

			LOD	LS	OB	MPR	NOD
**9 – 16**	56	10	0(0.00)	0(0.00)	0(0.00)	8(80.00)	2(20.00)
**17 – 24**	63	18	3(16.67)	0(0.00)	0(0.00)	11(61.11)	4(22.22)
**25 – 32**	68	27	10(37.04)	5(18.52)	0(0.00)	3(11.11)	9(33.33)
**33 – 40**	53	27	11(40.74)	9(33.33)	0(0.00)	1(3.70)	6(22.22)
**41 & above**	65	37	18(48.65)	10(27.03)	0(0.00)	0(0.00)	9(24.32)
**Total**	305	119	42(35.29)	24(20.17)	0(0.00)	23(19.33)	30(25.21)

LOD = Lichenified onchodermatitis, LS = Leopard skin, MPR = maculopapular rashes, NOD = Nodules, Onchocercal blindness = OB

This was followed by age group 25 – 32 years (37.04%). Palpable nodules were observed in all age groups with the highest (33.33%) prevalence recorded in age groups 25 – 32 years (Table 3). Onchocercal blindness was not recorded among any of the age groups. Responses from questionnaires revealed that among the villages, only 62 (43.36%) in Ogbozalla had the knowledge of the existence of onchocerciasis in Opi-Agu. The least perception of the diseases was observed in Idi village 48(36.36%) (Table 4).

**Table 4:** Villagers knowledge of the prevalence of onchocerciasis in Opi-Agu a tropical semi-urban community in Enugu State, Nigeria

Respondent	Number sampled	Responses			

		Yes	%	No	%
**Idi Village**	132	48	36.36	84	63.64
**Ogbozala Village**	143	62	43.36	81	56.64
**Ibeku Village**	135	56	41.48	79	58.52
**Total**	410	166	40.49	244	59.51

Majority of the inhabitants of Opi-Agu community (26.6%) believed that poor personal hygiene which ranked highest among other causes was the cause of onchocerciasis. This was followed by witchcraft (23.9%) and tsetse fly (22.7%), while bite of blackfly ranked least (14.9%) (Table 5).

**Table 5:** Villagers perception of the source or course of onchocerciasis in Opi-Agu a tropical semi-urban community in Enugu State, Nigeria

Respondent	Number sampled	Responses				

		Mosquito	Tsetse fly	Blackfly	Witchcraft	Poor hygiene
**Idi Village**	132	16(12.1)	18(13.6)	14(10.6)	38(28.8)	46(34.8)
**Ogbozala Village**	143	22(15.4)	39(27.3)	26(18.2)	31(21.7)	25(17.5)
**Ibeku Village**	135	11(8.2)	36(26.7)	21(15.6)	29(21.5)	38(28.1)
**Total**	410	49(11.9)	93(22.7)	61(14.9)	98(23.9)	109(26.6)

Number in parenthesis = percentage response

## Discussion

This study showed that Opi-Agu community is endemic for onchocerciasis. The overall prevalence of 39.02% is an indication that the disease is being actually transmitted in the community. The moderately higher prevalence observed in the older cohorts clearly explain the cumulative nature of the disease (16). Age groups 41 and above had higher prevalence (39.09%) than other age groups. Among the sexes the males (43.24%) were noted to have higher infection rate than the females (35.03%). This can be explained on the bases of occupational differences and degree of exposure to blackfly. In Opi-Agu community, while the males are normally involved in agricultural work in farms where they spend most of the hours of the day, the females are engage in petty trading in the local markets and this reduces the chances of contact with the disease vector (*S. damnosum*) among females. The males while cultivating usually expose their body and are more prone to infection due to bite of blackfly. Okoye and Onwuliri (17) also reported higher prevalence of onchocerciasis in the males among the inhabitants of Hawel River valley, Nigeria.

Blindness which is the most devastating effect of onchocerciasis (18) was not observed in Opi-Agu community but other signs such as lichenified onchodermatitis, leopard skin and palpable nodules (18–20) among others were observed****. Lichenified onchodermatitis was the most prevalent (35.29%) onchocercal sign. Among age group 9-16 years, leopard skin and lichenified onchodermatitis were entirely absent and palpable nodules ranked least (20%) while maculopapular rashes had the highest (80.00%) prevalence. The finding of this study was is in line with the findings of Okulicz et al. (4) and Okoye and Onwuliri (17) who reported the presence of lichenified onchodermatitis in older individuals, while it was absent in younger persons. According to WHO (5) onchocerciasis global burden was put at 987,000 disability adjusted life years (DALYs) and the severe pruritus alone accounted for 60% of the DALYs. Infection reduces the host's immunity and to other diseases, which may result in an estimated reduction in life expectancy of up to 13 years or more.

Questionnaire responses on knowledge of the disease and cause of infection revealed that many of the community members had poor knowledge of the disease, the vector and of its etiologic organism. The findings of this study points to the need for integration of community health education with mass chemotherapy. The African Program for Onchocerciasis Control (APOC) was established in Nigeria in December 1995 by WHO at the World Bank Headquarters in Washington DC, USA. APOCs strategy for the control of onchocerciasis was Community Directed Treatment with ivermectin (CDTI) aimed at effective and sustainable, community-directed treatment with ivermectin throughout the endemic areas within the geographic scope of the programme, and, if possible, eradication of the vector in selected and isolated foci, by using environmentally safe methods (5, 18).

## Conclusion

There is therefore an urgent need for CDTI intervention programme in Opi-Agu autonomous community, Enugu State, Nigeria as a means of controlling the spread of onchocerciasis in the communities and its immediate environs.
